# MSP22.8 is a protease inhibitor‐like protein involved in shell mineralization in the edible mussel *Mytilus galloprovincialis*

**DOI:** 10.1002/2211-5463.12286

**Published:** 2017-09-17

**Authors:** Juan Calvo‐Iglesias, Daniel Pérez‐Estévez, África González‐Fernández

**Affiliations:** ^1^ Immunology Biomedical Research Center (CINBIO) Centro Singular de investigación de Galicia Institute of Biomedical Research of Vigo (IBIV) University of Vigo Pontevedra Spain; ^2^ NanoImmunoTech Vigo Spain

**Keywords:** biomineralization, extrapallial fluid, mass spectrometry, shell matrix

## Abstract

The mussel shell protein 22.8 (MSP22.8) is recognized by a monoclonal antibody (M22.8) directed against larvae of the mussel *Mytilus galloprovincialis*. After being secreted by cells of the mantle‐edge epithelium into the extrapallial (EP) space (the gap between the mantle and the shell), the protein is detected in the extrapallial fluid (EPF) and EP hemocytes and finally becomes part of the shell matrix framework in adult specimens of *M. galloprovincialis*. In the work described here, we show how MSP22.8 is detected in EPF samples from different species of mussels (*M. galloprovincialis*,* Mytilus edulis*, and *Xenostrobus securis*), and also as a shell matrix protein in *M. galloprovincialis*,* Mytilus chilensis*, and *Perna canaliculus*. A multistep purification strategy was employed to isolate the protein from the EPF, which was then analyzed by mass spectrometry in order to identify it. The results indicate that MSP22.8 is a serpin‐like protein that has great similarity with the protease inhibitor‐like protein‐B1, reported previously for *Mytilus coruscus*. We suggest that MSP22.8 is part of a system offering protection from proteolysis during biomineralization and is also part of the innate immune system in mussels.

**Abbreviations**
ECM: extracellular matrix
EMs: electron micrographs
FA: focal adhesion
FAK: focal adhesion kinase
GEFs: guanine nucleotide exchange factors
iPALM: interferometric photoactivated localization microscopy
IRM: interference reflection microscopy

Biomineralization processes and biominerals have been studied for a number of years with the aim of fully understanding them and obtaining information to be applied in different fields of knowledge such as nanotechnology, bioscience, and even biomedicine 1, 2, 3, 4, 5. For these reasons, mollusks constitute one of the most widely studied groups of biomineral‐forming animals.

In mollusks, the central organ involved in shell formation is the mantle 6. Molluscan shell proteins are synthesized and secreted by cells of the mantle epithelium 1. According to the classical theory, secreted macromolecules are self‐assembled into the extrapallial (EP) space (the space between the mantle and the periostracum that covers the shell) without the participation of any cell 7. However, some authors defend the idea of juxtaposition of mantle cells that suggests a narrow contact between mantle cells and the mineralization site 5, 7, 8.

Nevertheless, in this scenario, there is no clear function for the extrapallial fluid (EPF). Although EPF is believed to play a major role in shell formation, there are very few studies that focus on this particular liquid or its proteins 9, 10. Most of these studies only concern the inorganic composition 11, 12, 13 or the macromolecules 14, 15, 16. However, EP proteins could participate either directly or indirectly in shell formation even if they are not present in the shell 17. In fact, this situation has been reported for two EP proteins 9, 10, 18. In addition to mantle cells, which play a central role in shell formation, EP hemocytes are also believed to participate in the biomineralization processes 7.

The identification and characterization of shell proteins was classically based on the isolation of individual proteins from the shell material in a ‘protein‐by‐protein’ approach. The development of methods based on proteomics or transcriptomics, or both, has enabled screening of shell proteins and this allows information to obtained on the ‘shellome’ 7. However, there is still a lack of genomic information for mollusks 19.

In previous publications, we described the generation of the monoclonal antibody (mAb) M22.8 20, 21 directed against *Mytilus galloprovincialis* larvae, which recognizes an antigen referred to as mussel shell protein 22.8 (MSP22.8) 22. We demonstrated that MSP22.8 is secreted by the mantle‐edge cells into the EP space, thus forming part of the EPF. We also showed how MSP22.8 is detected in EP‐resident hemocytes (invertebrate immune cells), but not in those from the hemolymph, and that hemocytes have the capability to take up the protein, with the MSP22.8 antigen finally being detected as a shell matrix protein 22.

In contrast to the situation described for other EP proteins 9, 10, 18, MSP22.8 constitutes the first individual EP protein that is also found as a shell matrix protein. In the work described here, we carried out a multistep purification strategy to isolate and identify MSP22.8 from EPF. A *de novo* sequencing‐assisted database search was employed together with a classical database search and it was concluded that MSP22.8 is a protease inhibitor‐like protein that has a strong similarity with A0A0K0YAZ2 (protease inhibitor‐like protein‐B1), which was previously described for *Mytilus coruscus*. In addition to *M. galloprovincialis*, we show how the protein is also detected in the EPF of *Mytilus edulis* and *Xenostrobus securis* and in the shell organic matrix of *Mytilus chilensis* and *Perna canaliculus*. The potential function of this protein in both biomineralization processes and the innate immune system is discussed.

## Results

### Concentration of the MSP22.8 antigen by ammonium sulfate precipitation

**Figure 1:**
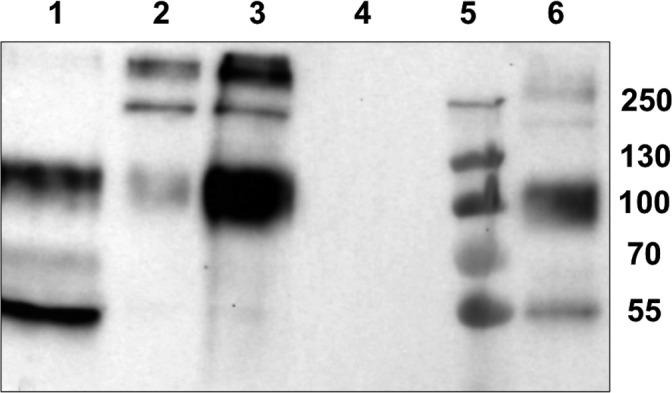
Western blot of EPF fractions obtained by AS precipitation. (1) Pellet obtained at 75% AS; (2) pellet obtained at 25% AS; (3) pellet obtained at 50% AS; (4) supernatant at 75% AS; (5) molecular mass markers; (6) EPF control.

MSP22.8 was previously described 22 as a mussel antigen that mainly consists of two bands: an upper band of about 100 kDa and a lower band of about 55 kDa. When the *M*. *galloprovincialis* EPF was subjected to ammonium sulfate (AS) precipitation, MSP22.8 was detected mainly in fractions corresponding to 50% and 75% AS (Fig. 1).

The upper band of MSP22.8 (100 kDa) was observed in all fractions, although the pellet obtained at 50% AS showed the strongest positive reaction (Fig. 1, Line 3).

The lower band of MSP22.8 (approximately 55 kDa) was only detected in the EPF precipitated with 75% AS. Bands corresponding to a molecular mass above 130 kDa were detected simultaneously in pellets obtained at 25% and 50% AS, which suggests the presence of multimers or aggregates of MSP22.8. Bands were not detected in the remaining supernatant after precipitation with 75% AS.

### Immunoaffinity chromatography

Given that the two intense bands of interest observed in the EPF fraction (the upper and the lower bands) were only present in the EPF fraction at 75% AS, this fraction was further purified using an affinity chromatography column. The column was prepared with mAb M22.8 in order to capture specifically the antigen MSP22.8.

**Figure 2:**
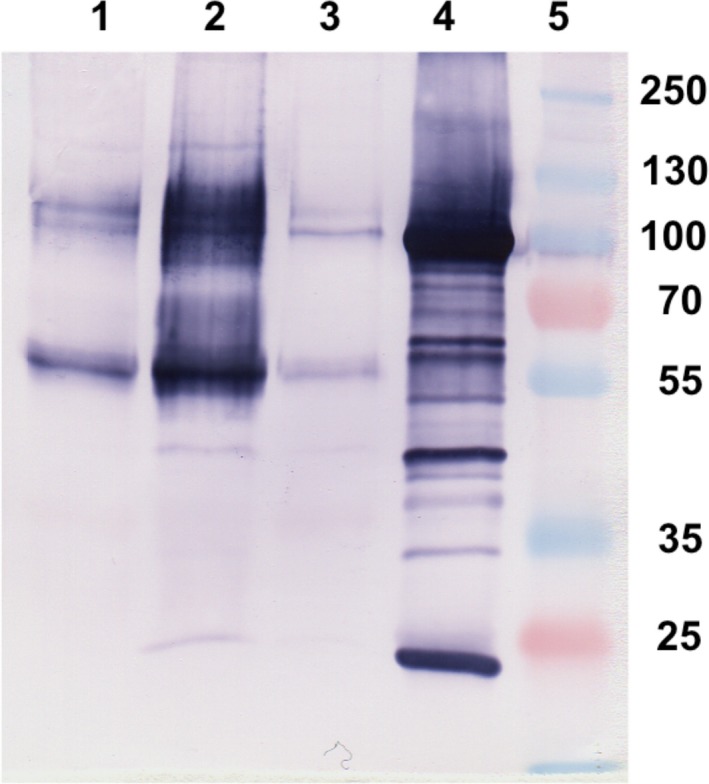
Immunoaffinity chromatography of the EPF fraction obtained at 75% AS. Colorimetric western blot after 10% SDS/PAGE (under reducing conditions). (1) EPF before passing through the column; (2) concentrated eluted fraction after immunoaffinity chromatography using mAb M22.8 in the column; (3) EPF wash; (4) M22.8 hybridoma supernatant; (5) molecular mass markers.

The eluted fractions were then evaluated by western blot (Fig. 2).

It can be seen in Fig. 2 that strong bands at 55 and 100 kDa appear to be concentrated in the purified fraction (lane 2), indicating that the mAb is able to capture both bands. Bands (called IC bands) from different experiments were manually excised from gels for further analysis by mass spectrometry.

### Protein purification by fast protein liquid chromatography (FPLC)

**Figure 3:**
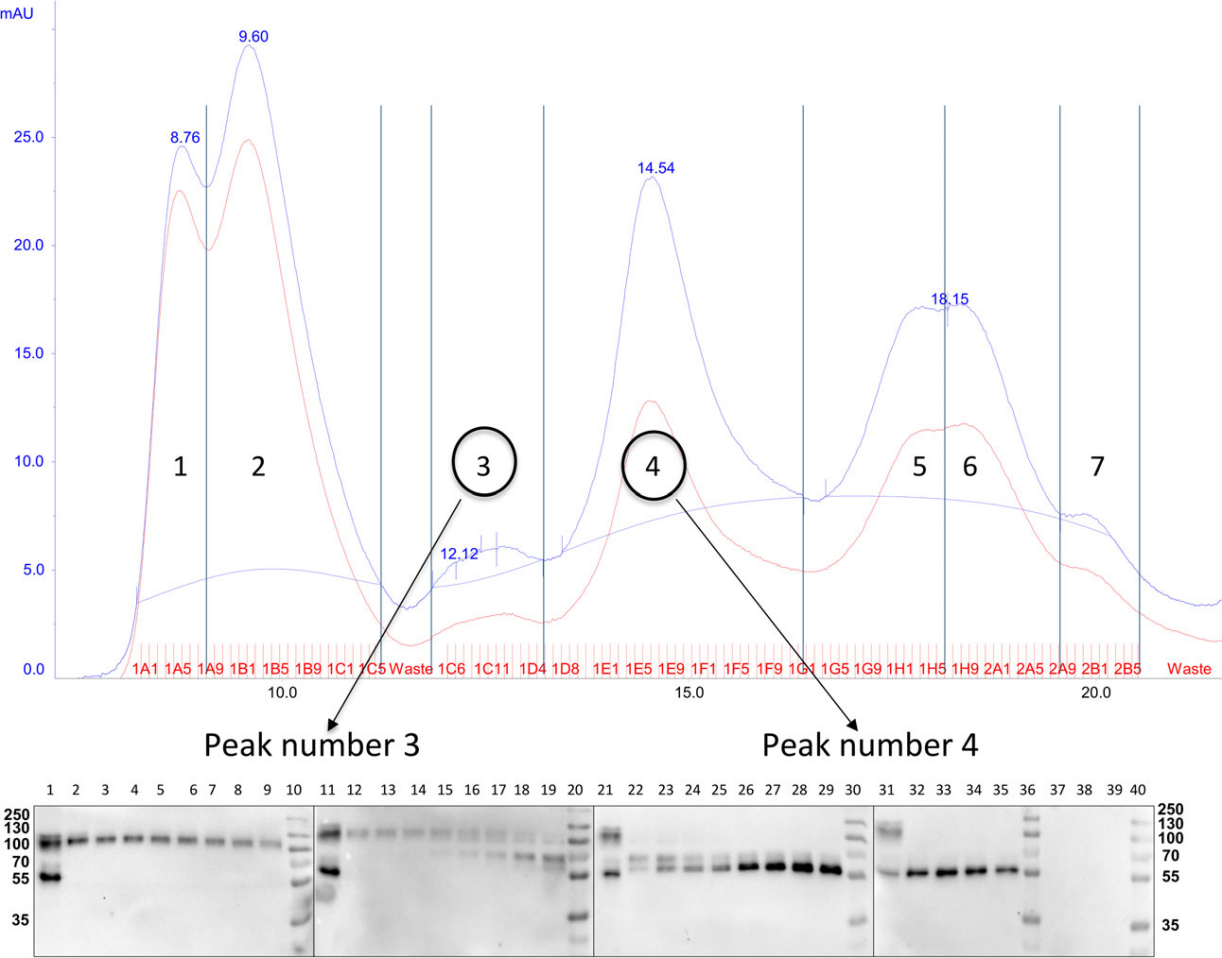
Chromatogram and western blot of EPF fractions obtained at 75% AS. Five microlitre of EPF fractions corresponding to peak numbers 3 and 4 was assayed by western blot after SDS/PAGE (10%) under reducing conditions. (mAU) milli absorption units.

Pellets obtained at 75% AS were also processed by FPLC. Eluates were checked by dot blot to confirm the presence of MSP22.8 (data not shown). Only those samples that tested positive were assayed by western blot. It can be seen in Fig. 3 that MSP22.8 was detected in two peaks, namely 3 and 4.

In peak number 3, the first 11 fractions only showed the band at 100 kDa. However, a band at 70 kDa started to appear in the transitional zone between peaks 3 and 4 (Fig. 3, western blot of fractions of peak number 3).

Peak number 4 showed two bands (around 55 and 70 kDa) in the first seven fractions, and thereafter, only the 55 kDa was band was visible in the last five fractions (Fig. 3, western blot of fractions of peak number 4).

**Figure 4:**
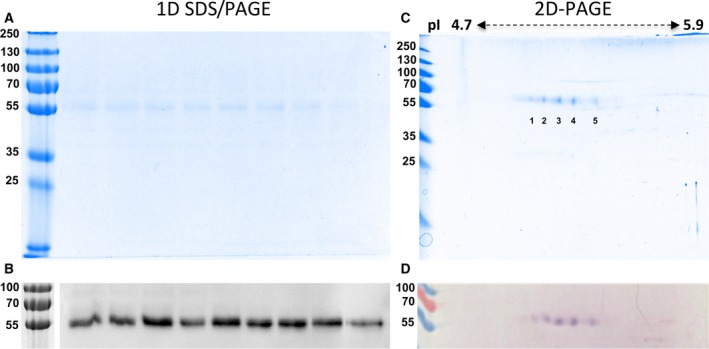
Electrophoresis and western blot of purified fractions. (A) 1D SDS/PAGE of purified fraction (5l) of EPF in 10% polyacrylamide minigel, Coomassie‐stained; (B) chemiluminescent western blot of the same fractions used in A; (C) 2D‐PAGE of purified fractions of EPF, using variations in the isoelectric point (pI) from 4.7 to 5.9, 10% polyacrylamide minigel, Coomassie‐stained; (D) colorimetric western blot of the same fractions used in (C).

Fractions corresponding to peak number 4 were further processed by 1D SDS/PAGE and 2D‐PAGE followed by western blot (Fig. 4). As shown in Fig. 4A, the assayed fractions mainly consisted of a single band at 55 kDa. This band was strongly positive under western blot assay (Fig. 4B).

The analysis by 2D‐PAGE, using different pI (from 4.7 to 5.9) on the same fractions, showed a train figure of five spots (Fig. 4C), all of which were positive in the western blot assay with the mAb M22.8 (Fig. 4D). These results suggest that MSP22.8 is a protein with several post‐translational modifications (PTM).

Bands and spots from different experiments were manually excised from gels and analyzed by mass spectrometry. Only central spots (namely spots 2, 3, and 4) were considered for further processing.

Fractions from peak number 3 (Fig. 3) were subjected to the same experiments as described above. However, the amount of protein was not sufficient to produce visible bands in SDS/PAGE experiments.

### Protein identification by mass spectrometry

Mass spectrometry was performed on the excised bands. MS/MS data were searched with Proteome Discoverer (SEQUEST) and Peaks software. *Mytilus* genus sequences available in Uniprot were used for the search along with a dataset of common contaminants (mammals + humans). A list of the top five scored protein candidates is provided in Table 1.

**Table 1:** Top scored proteins identified by peaks (Bioinformatics Solutions Inc., Waterloo, Canada) and proteome discoverer software (Thermo Fisher Scientific Inc., Waltham, MA, USA). IC band 55 and IC band 100 were purified by immunoaffinity chromatography (IC). The other bands (1.2, 2.2, 1.1, 2.1 and spots 2, 3, and 4) were isolated by FPLC. Protein identifications were sorted by SEQUEST HT score and Peaks protein score (−10lgP)

	Band 1.2	Band 2.2	Band 1.1	Band 2.1	Spot 2	Spot 3	Spot 4	IC band 55	IC band 100
Proteome Discoverer Sorted by SEQUEST HT score (…)	AKS48146.1	AKS48146.1	AKS48146.1	AKS48146.1	AKS48146.1	AKS48146.1	AKS48146.1	AFQ32467.1	CBX41655.1
	(771.72)	(756.92)	(315.98)	(721.51)	(18.19)	(323.25)	(49.81)	(122.19)	(5.88)
	AFM30917.1	AFM30917.1	AKS48147.1	AKS48147.1		AFM30917.1	AKS48147.1	AKS48146.1	AIL82396.1
	(117.44)	(89.94)	(124.53)	(183.22)		(65.74)	(7.95)	(28.21)	(5.88)
	AKS48147.1	AKS48147.1	AFM30917.1	BAJ07193.2		AKS48147.1	AFM30917.1	AKS48155.1	AAQ63463.1
	(98.93)	(54.47)	(12.21)	(40.91)		(13.27)	(6.83)	(6.37)	(5.88)
	CBX41746.1	AKS48186.1		AFM30917.1					AKS48146.1
	(35.94)	(38.20)		(31.75)					(4.95)
	AKS48186.1	CBX41746.1		AFQ32467.1					AKQ70857.1
	(34.40)	(30.88)		(1.73)					(1.66)
Peaks Sorted by −10lgP (…)	AKS48146.1	AKS48146.1	AKS48146.1	AKS48146.1	AKS48146.1	AKS48146.1	AKS48146.1	AFQ32467.1	AKS48146.1
	(600.53)	(584.66)	(362.52)	(358.52)	(266.92)	(393.4)	(252.38)	(525.74)	(236.54)
	AFM30917.1	AFM30917.1	AKS48147.1	AFM30917.1	AKS48155.1	AFM30917.1	AFM30917.1	AKS48146.1	AIL82396.1
	(484.56)	(472.66)	(252.78)	(253.23)	(136.09)	(375.40)	(225.43)	(387.72)	(184.53)
	AKS48147.1	AKS48147.1	AFM30917.1	AKS48147.1		AKS48147.1		AKS48147.1	AKQ70857.1
	(424.08)	(392.44)	(217.38)	(237.30)		(226.91)		(242.65)	(157.01)
	CBX41746.1	CBX41746.1		AKS48155.1				AKS48155.1	AKS48155.1
	(345.93)	(304.42)		(136.01)				(213.02)	(94.06)
	AKS48155.1	AKS48186.1		AKS48142.1					
	(334.02)	(302.15)		(116.48)					

Legend: AKS48146.1 protease inhibitor‐like protein‐B1 [*Mytilus coruscus*]; AFM30917.1 procollagen‐proline dioxygenase beta subunit [*Mytilus galloprovincialis*]; AKS48147.1 protease inhibitor‐like protein‐B2, partial [*Mytilus coruscus*]; AKS48155.1 protease inhibitor‐like protein‐B3, partial [*Mytilus coruscus*]; AKS48142.1| collagen‐like protein‐2 [*Mytilus coruscus*]; AIL82396.1 UNVERIFIED: elongation factor G, partial [*Mytilus trossulus*]; AKQ70857.1 nacre c1q domain‐containing protein 1, partial [*Mytilus galloprovincialis*]; AFQ32467.1 CuZn superoxide dismutase [*Mytilus galloprovincialis*]; CBX41746.1| putative C1q domain‐containing protein MgC1q97 [*Myttlus galloprovincialis*]; AKS48186.1 collagen‐like protein‐8, partial [*Mytilus galloprovincialis*]; AAQ63463.1 EP protein precursor [*Mytilus edulis*]; BAJ07193.2 estrogen receptor [*Mytilus galloprovincialis*]; CBX41655.1 putative C1q domain‐containing protein MgC1q6 [*Mytilus galloprovincialis*].

Bands are labeled as X.Y (1.1, 1.2, 2.1, and 2.2), and this denotes replicate (X) and experiment number (Y), respectively. All of the bands correspond to the FPLC fractions (peak 4) containing the lower band of MSP22.8 (55 kDa).

It can deduced from the results in Table 1 that a shell matrix protein reported for *M. coruscus* 23 known as protease inhibitor‐like protein‐B1 – henceforth referred to as PILP‐B1 (accession version AKS48146.1; Uniprot identifier A0A0K0YAZ2 (A0A0K0YAZ2_MYTCO) – is the top scored candidate detected in the FPLC‐processed samples. Moreover, there is almost complete agreement between the two software engines and this PILP‐B1 protein was also identified in samples obtained by IC.

Although other candidates, such as procollagen‐proline dioxygenase beta subunit (*M. galloprovincialis*) (AFM30917.1), were also identified in most FPLC samples, matches were not found in IC samples. This protein has been reported to have a MW of 55 094 kDa, which explains why this protein appears in the samples processed by FPLC, where the molecular mass is the key factor for isolation. However, the protein does not appear in the IC samples, where a column with a specific mAb was used. Thus, we can rule out this protein as our target antigen.

A list of the 25 detected peptides that support the identification of a PILP‐B1‐like protein is provided in Table 2. Only peptides identified by both software engines are shown in Table 2.

**Table 2:**
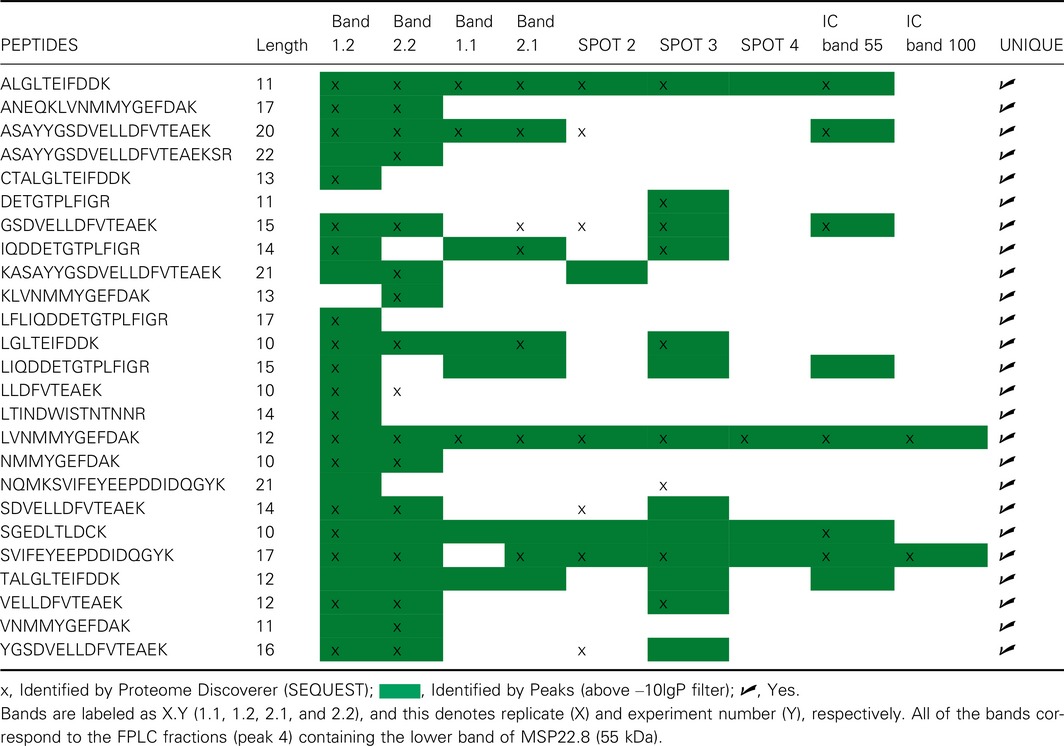
Detected peptides for PILP‐B1‐like protein identification. IC band 55 and IC band 100 were purified by immunoaffinity chromatography (IC). The other bands (1.2, 2.2, 1.1, 2.1 and spots 2, 3, and 4) were isolated by FPLC. Peptides were identified by both Proteome Discoverer (SEQUEST HT) and Peaks software engines (−10lgP) in at least one sample

Samples purified on a size‐exclusion column gave the highest number of identified peptides by both search engines. Although IC bands showed a lower number of peptides, the results obtained for these samples were crucial to identify the MSP22.8 antigen properly.

**Figure 5:**
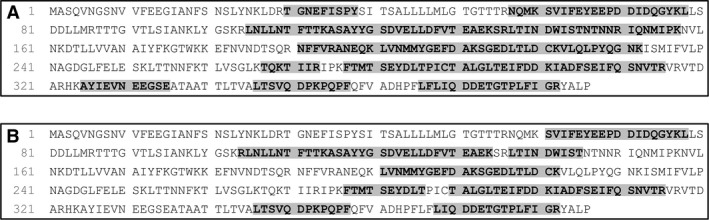
Coverage of peptides on the PILP‐B1 (AKS48146.1) in processed samples. (A) Coverage in an FPLC sample. (B) Coverage in an IC sample. Identified peptides are marked in bold and gray.

An example of the peptide coverage on the PILP‐B1 (AKS48146.1) is shown in Fig. 5. Samples of EPF purified by the FPLC approach produced better coverage than IC samples, thus ensuring that these samples gave more identified peptides.

### Protein identification by transcriptome analysis

An MS/MS search was performed against some available transcriptome data from *M. galloprovincialis* and *M. edulis*. The top scored identified hits were sent to BLAST (Nr database, restricted to genus *Mytilus*). As can be seen from the results in Table 3, only matches with an *E* value < 1e–10 were considered and reported.

**Table 3:**
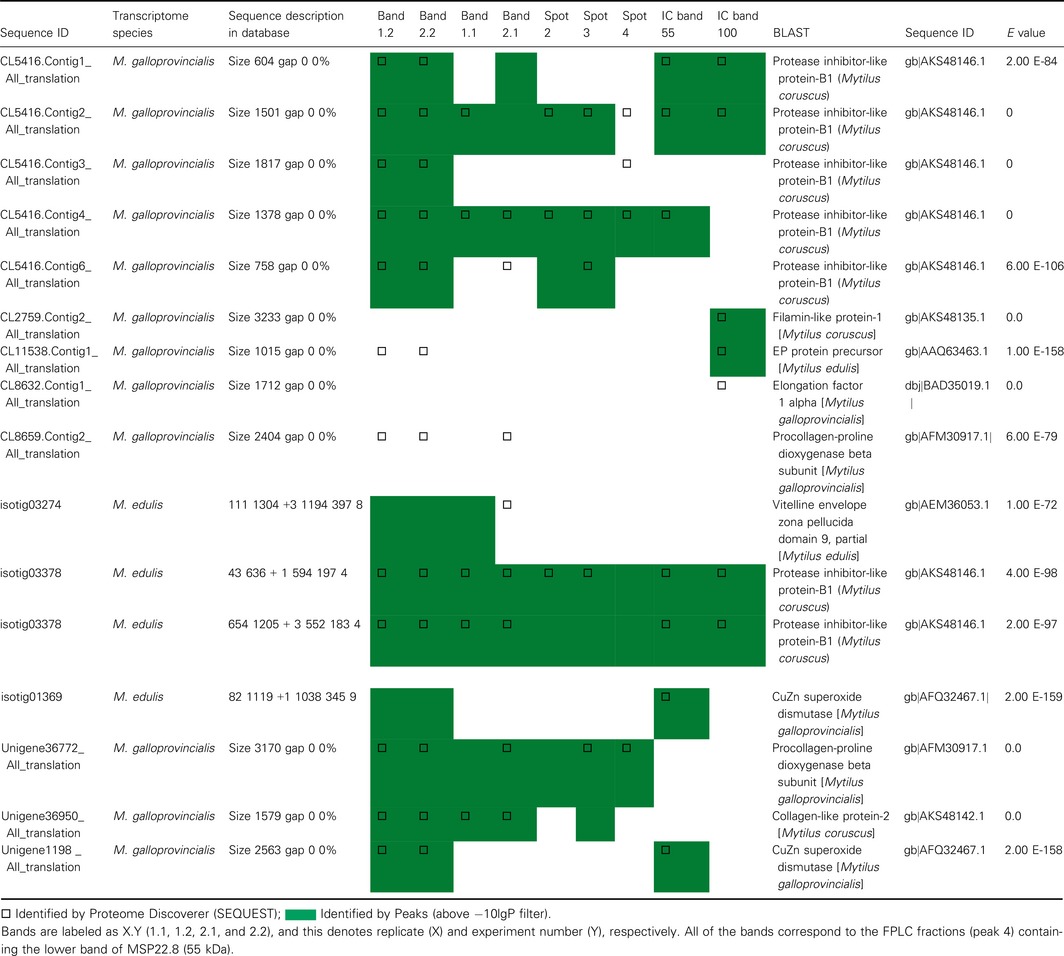
Top scored sequences identified in *M*. *galloprovincialis* and *M. edulis* transcriptome having significant BLAST results. Sequences were sorted on the basis of the SEQUEST HT (Proteome Discoverer) score and −10lgP (Peaks). The best matches were sent to BLAST. Only significant results (*E* value < 1e–10) are reported. IC band 55 and IC band 100 were purified by immunoaffinity chromatography (IC). The other bands (1.2, 2.2, 1.1, 2.1) and Spots (2, 3, and 4) were obtained by FPLC. Peptides were identified by both Proteome Discoverer (SEQUEST HT) and Peaks software engines (−10lgP), at least in one sample

**Figure 6:**
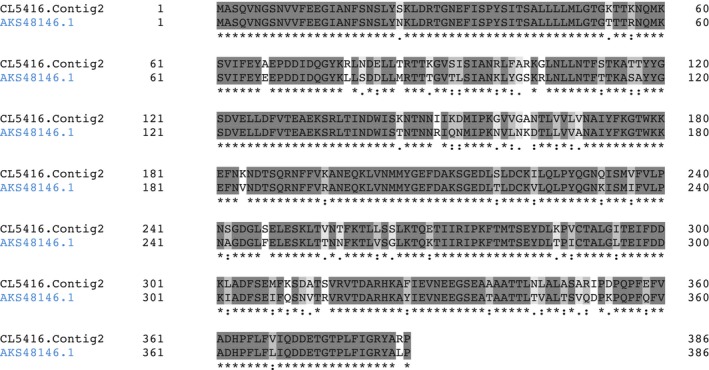
Alignment of PILP‐B1 (AKS48146.1) from *M. coruscus* and CL5416.Contig2 from *M*. *galloprovincialis*. Sequence related to CL5416.Contig2 refers to translation under frame +2. (*) indicates positions that have a single, fully conserved residue. (:) indicates conservation between groups with strongly similar properties. (.) indicates conservation between groups with weakly similar properties.

CL5416.Contig2 appears in most of the samples assayed (see Table 3). The BLAST result on this contig from *M. galloprovincialis* transcriptome showed a very high similarity (*E* value = 0) with PILP‐B1. Translation of the CL5416.Contig2 sequence under frame +2 contains an open reading frame (ORF), which shows strong sequence similarity (83.161% of identity) with PILB‐B1, as shown in Fig. 6.

The MS/MS search was also performed against *M. edulis* transcriptome, and the isotig03378 was found at the top in the score identification. A Blastp search of this sequence also showed a high similarity (*E* value = 1e–106) with PILP‐B1 (A0A0K0YAZ2).

**Figure 7:**
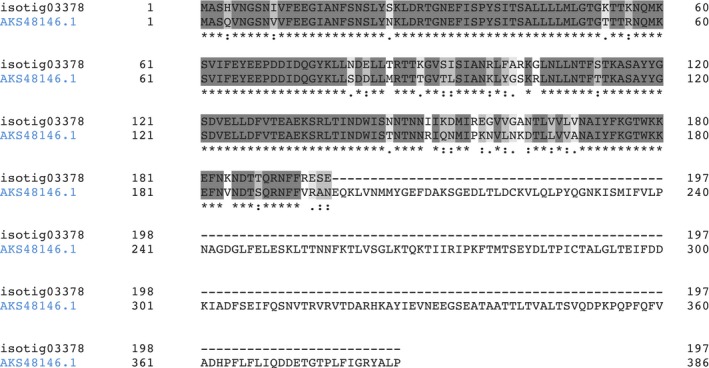
Alignment of PILP‐B1 (AKS48146.1) from *M. coruscus* and isotig03378 from *M. edulis*. (*) indicates positions that have a single, fully conserved residue. (:) indicates conservation between groups with strongly similar properties. (.) indicates conservation between groups with weakly similar properties.

The similarity of the two sequences is shown in Fig. 7.

**Figure 8:**
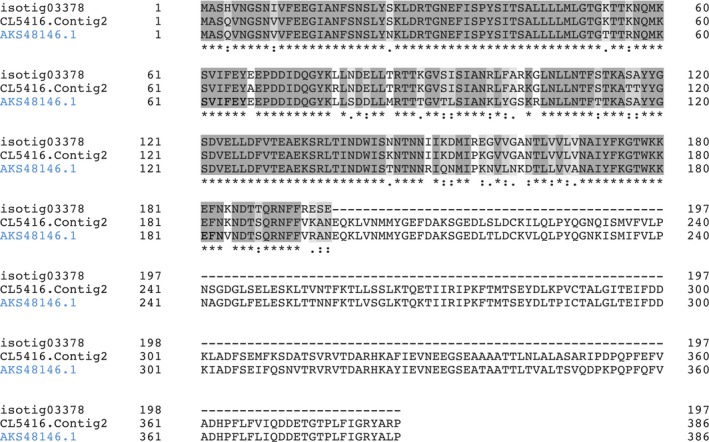
Alignment of PILP‐B1 (AKS48146.1) from *M. coruscus* with isotig03378 from *M. edulis* and CL5416.Contig2 from *M*. *galloprovincialis*. (*) indicates positions that have a single, fully conserved residue. (:) indicates conservation between groups with strongly similar properties. (.) indicates conservation between groups with weakly similar properties.

An alignment of the three sequences is shown in Fig. 8, in which the common residues are highlighted. If we consider the first 197 residues, the strongest similarity was found between isotig03378 (from *M. edulis*) and CL5416.Contig2 (from *M*. *galloprovincialis*) with around 93% of identity.

Moreover, CL5416.Contig2 had a higher similarity (83.25% of identity) with PILB1 (from *M. coruscus*) than isotig03378, with 81.22% similarity between the two sequences. However, it should be noted that isotig03378 from *M. edulis* gives partial information, because it may only refer to an incomplete or truncated form of PILB‐B1.

It can be concluded that the antigen recognized by mAb M22.8 is a protein that shows a strong similarity with the PILP‐B1 protein described in *M. coruscus*, but it is not identical. More marked similarities were found between *M*. *galloprovincialis* and *M. edulis* in this protein than with that in *M. coruscus*.

### Detection of MSP22.8 in the EPF of different mussel species

With the aim of clarifying whether MSP22.8 appears in the EPF of species other than *M. galloprovincialis*, fresh EPF from *X. securis* and *M. edulis* was assayed.

**Figure 9:**
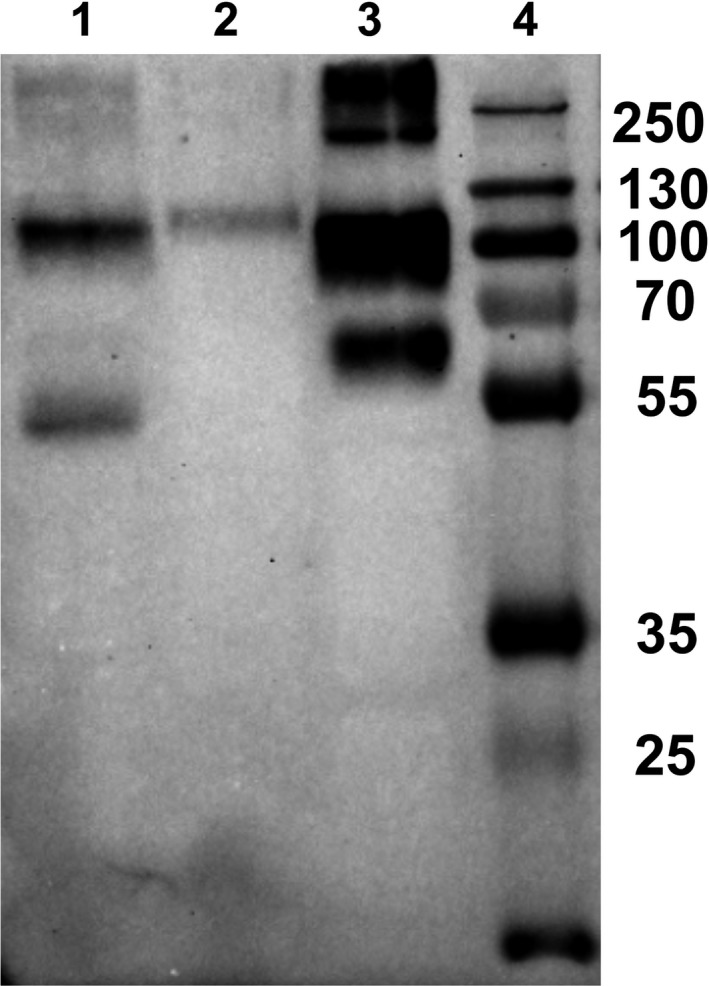
Western blot assay on EPF of different mussel species. (1) EPF of *M*. *galloprovincialis*; (2) EPF of *X. securis*; (3) EPF of *M. edulis*; (4) molecular mass markers (kDa). Ten microlitre of EPF was loaded on each lane.

As can be seen in Fig. 9, MSP22.8 was found in the EPF of all three species assayed, although differences in the electrophoretic pattern were detected. In *M. edulis*, the lower band showed an apparent molecular mass (approximately 65 kDa) higher than the counterpart (55 kDa) in *M*. *galloprovincialis*. Moreover, in *X. securis*, the upper band was weak and the lower band was not detected under the same conditions, thus suggesting that the *X. securis* samples contain a lower concentration of MSP22.8 as the same EPF volume was used in all cases.

### Detection of MSP22.8 in the shell organic matrix of different species of mussels

**Figure 10:**
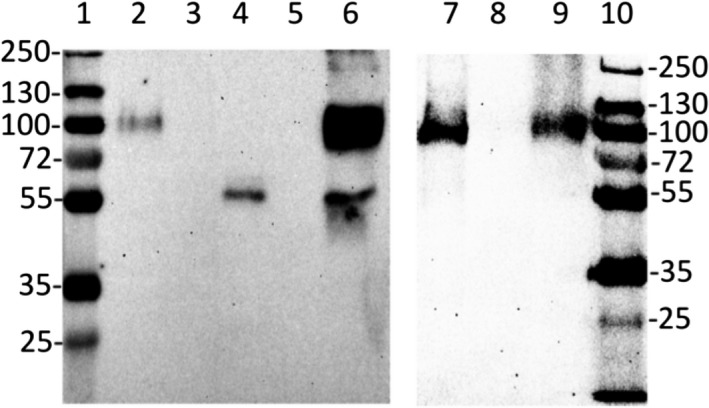
Western blot of shell samples of different mussels. (1 and 10) Molecular mass markers (kDa), (2) *M*. *galloprovincialis*, (3) empty well, (4) *M. chilensis*, (5) empty well, (6) EPF control, (7) *M*. *galloprovincialis*, (8) empty well, (9) *P. canaliculus*.

As an alternative to EPF, EDTA‐insoluble samples of shell organic matrix from *M. chilensis* and *P. canaliculus* (species from which fresh EPF was not locally available) were assayed. It can be seen from Fig. 10 that MSP22.8 was detected in both species.

The upper band of MSP22.8 was detected in EDTA‐insoluble samples of *M*. *galloprovincialis* and *P. canaliculus*, while the lower band was only detected in samples of *M. chilensis*. As reported previously 22, MSP22.8 appeared as an upper band and a lower band in shell samples of *M*. *galloprovincialis*. The disparity in these results could be explained by the nature of the samples (available samples of *M. chilensis* were cooked frozen samples of mussels), the complexity of sample decalcification with EDTA, and its tendency to produce nonremovable complexes with organic materials 24, thus making it very challenging to work with these samples. Another potentially important factor is the serpin‐like nature of MSP22.8. Serpins are proteins that are known to exhibit polymorphism 25.

### Detection of an ester bond

MSP22.8 showed an apparent dimeric nature under SDS/PAGE conditions. However, we have demonstrated that, under standard reducing conditions, the upper band exists together with the lower band 22. Serpins are proteins that are known to form stable complexes under standard SDS/PAGE conditions 26, 27. These proteins can form ester bonds 28 but they also tend to show polymorphism, thus forming stable complexes.

**Figure 11:**
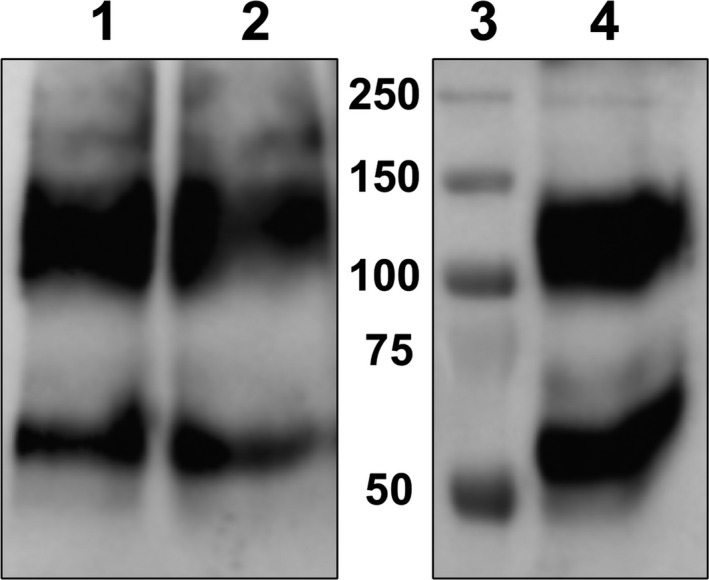
Western blot of alkaline‐treated samples. (1 and 2) Samples treated with ethanolamine, (3) molecular mass markers (kDa), (4) EPF control.

In an effort to clarify whether the presence of an ester bond could explain the differences observed, fresh samples of raw *M*. *galloprovincialis* EPF were subjected to an alkaline treatment with ethanolamine. This treatment was reported 29 to allow separation between a serpin and its protease. As shown in Fig. 11, samples pretreated with ethanolamine showed the same profile as control samples, which suggests that ester bond formation does not occur in this protein. The absence of an ester bond is in good agreement with the fact that a potential protease candidate was not identified within IC samples. According to our results, the nature of the upper band of MSP22.8 can be explained by the tendency of serpin to polymorphism.

## Discussion

In the present work, MSP22.8 from EPF of *M*. *galloprovincialis* was isolated using a multistep strategy involving AS fractionation followed by immunoaffinity chromatography (IC samples) or size‐exclusion chromatography (FPLC samples).

Eluted samples were further fractionated by SDS/PAGE or 2D‐PAGE and analyzed by LC‐MS/MS. MS/MS searches were carried out using two different software engines: The MS/MS spectra were searched using a classical MS/MS database search engine (SEQUEST), and the same spectra were searched with a *de novo*‐assisted database search engine (Peaks).

MSP22.8 was identified as a protease inhibitor‐like protein in all samples by both software engines. Moreover, the results are totally comparable, particularly in FPLC samples. MSP22.8 showed a great similarity (if not the same) with PILP‐B1, an analogous protein previously reported in *M. coruscus* 23. In addition to the above, MS/MS searches on transcriptome data for *M*. *galloprovincialis* and *M. edulis* identified sequences with a very high similarity with PILP‐B1. Overall, these results suggest that MSP22.8 has a similar (but not the same) sequence to that reported for the shell matrix protein described for *M. coruscus*.

The use of a double purification strategy allowed us to rule out candidates from the results. For example, *procollagen‐proline dioxygenase* (beta subunit) was discarded as a possibility. On the basis of peptide identification, the presence of this protein would be detected in most FPLC samples. However, IC samples were completely negative as peptides that resemble the protein were not identified in any case. Moreover, the protein in question was reported to be present in foot 30 and more recently in gills 31, but MSP22.8 was not detected in any of these tissues 22.

PILP‐B1 from *M. coruscus* has a putative serpin domain that belongs to the serine protease inhibitor (serpin) family. Serpins are a family of proteins that can function as serine proteinase inhibitors 32, 33. They are widely distributed and show a highly conserved structure and sequence 34. Although homology between members of the serpin family is poor, they do have a conserved core structure 35. They also exhibit a characteristic conformational polymorphism that shifts from native to cleaved, latent, delta, or polymorphic forms 25.

We believe that the capability of serpins to conform homodimers would explain the electrophoretical appearance of MSP22.8, thus suggesting that the protein exists as a 55‐kDa monomer, with the other forms being dimeric (100 kDa) and multimeric (> 130 kDa) species of the same protein. These high molecular mass bands did not produce clear visible bands upon staining the gels, perhaps due to the particularly poor staining in SDS/PAGE gels shown by shell matrix proteins 36.

Additionally, the nature of these bands could be explained if we assumed the serpin‐like nature of MSP22.8. It was reported that the polymeric form of serpins produces strong results in western blot due to epitope magnification 37. This characteristic would explain why clear bands on western blot do not have the corresponding images in SDS/PAGE gels. The fact that proteins involved in shell formation are not particularly well stained with the current staining methods (such as Coomassie or silver stain) exacerbates this problem. This would explain why the number of peptides derived from IC band 100 was particularly low. Despite the fact that bands were clearly detected in western blot assays, the bands looked reasonably weak when the conventional staining was performed.

The upper band (≈ 100 kDa) was the predominant one in most samples. The fact that this band appears as a doublet of the lower band (≈ 55 kDa) led us to explore first the possibility of dimer formation due to S–S bridges. However, the same pattern appeared under both reducing/nonreducing SDS/PAGE conditions 22. As a result, the behavior of MSP22.8 cannot simply be explained on the basis of the existence of disulfide bonds. Another possible explanation would be that the monoclonal antibody M22.8 could recognize a shared epitope by two different proteins. Despite the addition of urea, neither Dithiothreitol (DTT) nor beta‐mercaptoethanol was able to modify the upper band, but peptides that allow the identification of PILP‐B1 were found in both the upper (100 kDa) and lower (55 kDa) band (IC samples), which strongly suggests that the same protein is present in both bands as they are recognized by the mAb. Unfortunately, the mechanism of serpin dimerization/polymerization is not known 35, 38 but it is believed that this process occurs due to a domain swap mechanism 39.

Nevertheless, the particular pattern of MSP22.8 could be explained by the special behavior exhibited by serpins. It has been described how these proteins can act as suicide protease inhibitors by forming a stable covalent complex with proteases through ester bridge formation. This possibility was explored but significant changes were not observed in the MSP22.8 pattern in western blot after alkaline treatment. Additionally, the exploration of peptides identified by LC‐MS/MS in IC samples did not show any potential protease candidate.

Polymorphism of serpin could explain the electrophoretic pattern observed in MSP22.8. It has been reported that serpin can form stable dimers *in vitro* in the presence of SDS 40, 41, and dimer formation may be enhanced by low pH, denaturing agents, and heating 42, 43, 44. Although the aforementioned factors may affect dimer formation, in our experiments it was observed that dimerization would be produced before the SDS/PAGE procedure. In fact, samples containing the lower band of MSP22.8 did not dimerize in SDS/PAGE assays. Putative dimerization of MSP22.8 would occur naturally and/or due to unknown factors. The characteristic electrophoretical behavior can be explained on the basis of the serpin‐like nature of the protein.

Multiple epitope recognition or cross‐reaction 45 might explain the multiband pattern observed in western blot. However, polyreactivity and nonspecific binding are much more frequent when using polyclonal antibodies, especially if they contain IgM (due to its high valence) 46. Monoclonal antibodies are monospecific and are much less likely to cross‐react 47, particularly if they are of the IgG isotype 48, 49, like AcM M22.8. We consider that the multiband pattern is well explained by the presence of MSP22.8 in several forms (monomeric, dimeric, and polymeric forms). In fact, the proteomic analysis identified common peptides from the same protein in the B55 and B100 bands (obtained by immunoaffinity chromatography), and also in the B55 spots, which indicates that the lower band would correspond to the monomer form and the upper band to the dimeric form of the same protein. Proteomic analysis identified MSP22.8 as a serpin‐like protein. Serpins belong to a group of proteins characterized by their high tendency to show polymorphism and by giving signal magnification in the western blot technique.

MSP22.8 was detected in EPF from *M. edulis* and *X. securis*, both of which are considered as nonindigenous species in Galician waters 50, 51. Electrophoretically some differences were found and these could be due to PTM or sugar moieties. In fact, serpins are mostly glycoproteins 34. Indeed, the apparent molecular mass of MSP22.8 (≈ 55 kDa) would be higher than the theoretical value reported for PILP‐B1 (≈ 43 kDa).

MSP22.8 was also detected in the shell organic matrix in samples of *P. canaliculus* and *M. chilensis* subjected to EDTA demineralization. Decalcification with EDTA or acetic acid is a common method for shell decalcification 5. In the preliminary test, an acidic treatment was applied to shell samples but this proved to be unsuccessful. Although shell proteins could be extracted with the acid approach, MSP22.8 could not be detected in the acidic extracts. Hydrolysis of proteins is supposed to be moderate or absent during acetic acid decalcification 24. However, the acidic treatment could have a negative effect on antibody recognition 52 (e.g., by affecting epitope structure). This possible side effect was not found with the EDTA method, and this procedure was therefore selected. Several authors reported that EDTA treatment produced complexes with organic shell material that were difficult or impossible to resolve 5, 24. The nature of the samples, EDTA treatment, and the serpin‐like nature of MSP22.8 may explain the different pattern observed.

Detection of MSP22.8 in shell samples was an extremely challenging task, including the sourcing and prior processing of the shell samples (frozen, boiled, fresh), the complex process of demineralization, and the particular nature of MSP22.8 as a serpin‐like protein. Moreover, we cannot rule out the possibility that the differences found could also be due to multiple processing during shell formation. We have previously reported that the antibody is able to recognize the protein in the *M*. *galloprovincialis* shell 22, and we show here that it can also recognize it in other species.

The existence of protease inhibitor‐like proteins within the shell organic matrix is not new in mollusk 23, 53, 54, including serpin‐like proteins 23, 55. Proteinase inhibitors were found to be highly specific and abundant in shells in *Crassostrea gigas* 55. Interestingly, the expression of serpins was studied in Manilla clams (*Ruditapes philippinarum*) 56 and serpin expression was only detected in mantle cells, with negative results observed on hemocytes. These results are in good agreement with our findings. We have described how MSP22.8 is expressed and secreted by cells of the mantle edge, while negative results were obtained in hemolymph hemocytes. Moreover, it was demonstrated how hemocytes are able to internalize MSP22.8 when exposed to a cell‐free EPF solution 22. MSP22.8 was identified as a serpin‐like protein, and high similarity was found with sequences derived from transcriptome of *M*. *galloprovincialis* and *M. edulis*.

The expression of the sequence that resembles MSP22.8 in *M*. *galloprovincialis* transcriptome was reported in cells of the mantle tissue, while expression in stimulated hemocytes was found to be negative or very weak 57. According to our results, MSP22.8 is not detected in hemolymph hemocytes 22. Although our paper does not focus on the similarities or differences between the EPF and hemolymph, analysis of both samples gave a similar pattern in the acrylamide gels (1D SDS/PAGE). However, we have clearly demonstrated that the MSP22.8 antigen is not detected in hemolymph, but it is present in the EPF. Thus, we can say that both fluids differ at least in that protein. This circumstance situation is applicable to other mantle‐secreted proteins. If MSP22.8 is produced, the amount is insufficient to be detected by the monoclonal antibody M22.8 under the conditions studied. These findings are consistent with results obtained in the clam *R. philippinarum*, where serpin expression was found only in mantle cells but in hemocytes it was negative 56.

It has been suggested that protease inhibitors are involved in biomineralization processes as part of a protection system against proteolysis during shell formation 58, 59. Additionally, serine protease inhibitors are believed to be part of the innate immune system in invertebrates 60. The MSP22.8 reported here could be part of both systems. On the one hand, the protein may participate in a protective system against protein degradation, as well as being part of the shell matrix framework. On the other hand, the protein may be involved in the innate immune system. In fact, it was suggested that the presence of protease inhibitors in the molluscan shell would protect the structure from enzymes secreted by fouling organisms and predators 61.

## Materials and methods

### Ethics statement

Permits were not required for the study, which complied with all relevant regulations.

### Mussels

Adult specimens of *M*. *galloprovincialis* purchased from a local seafood market (Flipper fish shop, Mercado do Berbés, Vigo, Spain) were collected from aquaculture populations of Galician bays, ría de Vigo (42°15′N 8°45′W). Adult specimens of *M. edulis* were purchased in a hypermarket (Auchan hypermarket, Vigo, Spain) and were collected from aquaculture populations in France. Adult specimens of *X. securis* were collected from wild populations of the ría de Vigo (42°20′44.3″N 8°36′44.3″W).

Unless specifically described elsewhere, samples were obtained from a minimum of 10 individuals. Only healthy live animals were sampled. All experiments were performed at least three times including two replicates of each sample.

### Extraction and fractionation of extrapallial fluid (EPF)

Extrapallial fluid from adult mussels was extracted by inserting a needle coupled to a sterile syringe into the EP space. Punction was carried out carefully in order to avoid contact with the mantle surface and to avoid contamination with water or tissue debris. EPF was pooled, filtered through 0.45‐μm and 0.22‐μm filters, immediately transferred to 1.5‐mL sterile centrifuge tubes, and kept on ice. Centrifugation was performed at 16 000 ***g*** for 10 min at 4 °C. The supernatant (4 mL) was retained and kept on ice. Protein fractions of EPF from *M*. *galloprovincialis* were obtained by AS precipitation 62. An on‐line calculator from EnCor Biotechnology Inc. (http://www.encorbio.com/protocols/AM-SO4.htm) was employed to determine the amounts of solid AS required to reach a given saturation. Pellets obtained at 25%, 50%, 75%, and 100% AS were stored at −20 °C. A dialysis step against PBS in an ultrafiltration column (Amicon^®^ Ultra‐15; Merck Millipore Ltd., Tullagreen, Ireland) was included to remove AS salt.

### Shell material

Fresh shells (*M*. *galloprovincialis*) from local producers, frozen shells (*P. canaliculus*), and cooked frozen shells (*M. chilensis*), imported and purchased in a local market, were processed as reported previously 22. Adult shells were extensively brushed to remove epibionts and were soaked in 3% NaOCl for 1 h, rinsed with distilled water, and dried. This treatment allowed partial removal of the periostracum. Clean shells were crushed to give a powder. Shell powder was completely demineralized with 0.5 m EDTA (pH 7.8) in a dialysis cassette (Slide‐A‐Lyzer^®^ Dialysis Cassette; Thermo Fisher Scientific Inc.). After demineralization, EDTA‐soluble fractions were dialyzed against sodium phosphate buffer (PBS) and desalted with a centrifugal filter (Amicon Ultra‐0.5 Centrifugal Filter; Merck Millipore Ltd.). EDTA‐insoluble fractions were extensively washed with ultrapure water. The two EDTA fractions were lyophilized and stored at −20 °C prior to use. EDTA‐soluble fractions were rehydrated with Laemmli sample buffer (Bio‐Rad Laboratories, Inc.). Insoluble lyophilized samples were rehydrated with lysis buffer (urea 7 m, thiourea 2 m, CHAPS 4% w/v, DTT 3% w/v). The rehydrated samples were further precipitated with a commercial kit to remove contaminants (ReadyPrep™ 2‐D Cleanup Kit; Bio‐Rad Laboratories, Inc.). Protein pellets were finally resuspended by adding an appropriate volume of Laemmli sample buffer.

### Protein purification

#### Immunoaffinity chromatography

In order to purify EPF fractions, M22.8 monoclonal antibody was used as a ligand for an affinity chromatography column. Briefly, 0.6 mg of M22.8 antibody was covalently coupled to a 1‐mL HiTrap™ NHS‐activated HP column (GE Healthcare, Chigago, IL, USA). The column was manually operated with a syringe. Preparation, ligand coupling, washing, and deactivation steps were strictly performed in accordance with the manufacturer's instructions. Four millilitre of EPF was precipitated with AS. Pellets obtained at 75% AS were desalted and incubated with the column. Purification steps were performed according to the manufacturer's guidelines, using 0.2 m glycine/HCl pH 2.5 as elution buffer. Purified fractions were collected in clean Eppendorf tubes containing neutralization buffer (1 m Tris/HCl pH 9.0) and were desalted and concentrated on an Amicon^®^ Ultra 30K ultrafiltration column (Merck Millipore Ltd.).

#### Size‐exclusion chromatography

Pellets obtained at 50–75% AS were desalted and further fractionated on a size‐exclusion column (Superdex 10/300 GL; GE Healthcare, Little Chalfont, UK) coupled to a Fast Protein Liquid Chromatography system (Äkta purifier 10; GE Healthcare, UK). PBS 1 × was used as the equilibration and elution buffer. The flow rate was maintained at 0.5 mL·min^−1^. After equilibrating the column and stabilizing the signal at 280 nm, 500 μL of sample was injected. Eluted samples were collected according to their molecular size and tested by dot blot in order to confirm positivity.

#### Protein electrophoresis

Electrophoresis experiments (first dimension in SDS/PAGE; second dimension in 2D‐PAGE) were carried out using a Mini‐PROTEAN^®^ 3 or a Mini‐PROTEAN^®^ Tetra cell electrophoresis unit (Bio‐Rad Laboratories, Inc.). Isoelectric focusing on 2D‐PAGE was carried out in a PROTEAN^®^ IEF system (Bio‐Rad Laboratories, Inc.) using 11‐cm strips, pH 4.7–5.9 (ReadyStrip™ IPG Strip, Bio‐Rad Laboratories, Inc.). The protocols (SDS/PAGE and 2D‐PAGE) were carried out according to the manufacturer's instructions. Reagents of electrophoresis grade were obtained from Bio‐Rad Laboratories Inc., electrophoresis‐grade ammonium persulfate was purchased from Sigma Aldrich (St. Louis, MO, USA), and 1,2‐bis(dimethylamino)ethane was obtained from Merck Millipore Ltd. A broad range of molecular mass markers were purchased from Thermo Fisher Scientific Inc (Scientific PageRuler Plus Prestained Protein Ladder). Unless stated otherwise, samples were prepared following the Laemmli protocol 35. DTT (Bio‐rad Laboratories Inc.) was selected as the reducing agent. DTT was added to a final 1 × concentration of 50 mm. Samples were run on 10% SDS/PAGE vertical minigels under both nonreducing/reducing conditions (DTT included). In order to investigate the presence of an ester bond, samples of EPF obtained from 15 mussels were pooled, filtered, and then divided into two fractions, with one of the fractions treated with ethanolamine 29. Briefly, 300 μL of EPF was mixed with a solution of 270 μL of PBS and 30 μL of 2 mol·L^−1^ ethanolamine, and the sample was incubated at 25 °C for 24 h. The second fraction was processed in the same manner, except that ethanolamine was not added. This sample was used as control. Aliquots from both fractions were routinely processed by protein electrophoresis, as described above. Electrophoretic conditions were 200 V at constant current according to the manufacturer's instructions. Minigels were stained with Bio‐safe Coomassie stain (Bio‐Rad Laboratories, Inc.).

### Western blot and dot blot

Western blot assays 36 were carried out using minigels 37 transferred to PVDF membranes (Immun‐Blot^®^; Bio‐Rad Laboratories Inc.) using a Trans‐Blot^®^ Turbo™Transfer System (Bio‐Rad Laboratories Inc.). Transfer conditions were 25 V for 30 min. Membranes were washed in Tris‐buffered saline with 1% Tween 20 (TBST) and blocked with 5% skimmed milk (Sigma Aldrich Co.) in TBST overnight. After blocking, membranes were washed three times with TBST and incubated for 2 h with M22.8 hybridoma supernatant (1 : 10 dilution in TBST with 2.5% skimmed milk) at room temperature (RT). For colorimetric western blotting assays, goat anti‐mouse IgG antibodies conjugated to alkaline phosphatase (Dako, Glostrup, Denmark) diluted 1 : 1000 in TBST with 2.5% skimmed milk were used as secondary antibodies (1.5 h, RT). Goat anti‐mouse IgG antibodies conjugated to horse rabbit peroxidase (Dako) diluted 1 : 50 000 in TBST with 2.5% skimmed milk were used as secondary antibodies (1 h, RT) for chemiluminescence western blot assays. Colorimetric western blot assays were revealed with 1‐Step NBT/BCIP (Thermo Fisher Scientific Inc.), while the Immun‐Star™ WesternC™ Chemiluminescent Kit (Bio‐Rad Laboratories Inc.) was selected for chemiluminescence assays. Protein bands were analyzed using the ChemiDoc XRS imaging system in conjunction with imagelab software (Bio‐Rad Laboratories Inc.).

Dot blotting was performed in PVDF membranes (Immun‐Blot^®^; Bio‐Rad Laboratories Inc.) following the manufacturer's instructions. Briefly, 25 μL of sample was directly spotted on the wet membrane and allowed to dry. The other steps were performed as described above for western blotting.

### Mass spectrometry analysis

Bands and spots were in‐gel‐digested with trypsin (Promega, Madison, WI, USA) following the standard procedures. Briefly, gel slices were washed with water and 25 mm ammonium bicarbonate pH 7.8, and 25 mm ammonium bicarbonate pH 7.8 50% acetonitrile, reduced with 10 mm DTT, alkylated with 55 mm iodoacetamide, and digested with trypsin overnight at 37 °C. Tryptic peptides were extracted with 0.5% TFA and twice with acetonitrile. The combined extracts were evaporated to dryness.

The samples were analyzed by LC‐MS/MS using a high‐resolution LTQ/Orbitrap mass spectrometer equipped with an EASY SPRAY ion source (Thermo Fisher Scientific Inc.). Each extract was diluted to 12 μL with 0.1% formic acid. Ten microlitre samples was loaded onto a chromatographic system consisting in a C18 preconcentration column (Acclaim PepMap 100 75 μm × 2 cm; Thermo Fisher Scientific Inc.) connected to a 50‐cm‐long, 75‐μm i.d. PepMap C18 column (Thermo Fisher Scientific Inc.). The separation was performed at 0.3 μL·min^−1^ in an acetonitrile gradient (240 min) from 5 to 25% (solvent A: 0.1% formic acid, solvent B: acetonitrile 0.1% formic acid). The HPLC system was an EASY nLC‐1000 (Thermo Fisher Scientific Inc.). The Orbitrap instrument was operated in the positive ion mode with a spray voltage of 1.8–2 kV. The scan range of each full MS was *m*/*z* 400–1700. The spectrometric analysis was performed in an automatic‐dependent mode, acquiring a full scan and 15 MS/MS of the most abundant signals. Both MS and dependent MSMS scans were performed in the FT mode to maximize the mass resolution and precision of the MS. Dynamic exclusion was set to 1 to avoid the redundant selection of precursor ions.

### Bioinformatic analysis

MS/MS data were searched using SEQUEST (Proteome Discoverer 2.1; Thermo Fisher Scientific Inc.) with the following parameters: peptide mass tolerance 8 p.p.m., fragment tolerance 0.02 Da, enzyme set as trypsin or semitrypsin and allowance up to two missed cleavages, static modification was carbamidomethylated cysteine (+57 Da), and dynamic modification was methionine oxidation (+16 Da). The Uniprot database (taxonomy: *Mytilus*) together with a contaminant database (taxonomy: mammals + humans) was used for searching. At least two unique peptides were required for protein identification. Any sequence tag related to probable contamination (e.g., keratin and trypsin) was rejected and was not considered for further processing.

The chromatograms were reanalyzed using the PEAKS Studio search engine tools (PEAKS Studio 7.5; Bioinformatics Solutions Inc.). The search parameters were as follows: mass tolerance 8 p.p.m. for precursor ions and 0.02 Da for fragment ions. Searches were performed against the same databases as described above. The result filtering parameters were as follows: FDR 0.1% for peptide identification and at least two unique peptides per protein identification.

Additionally, MS/MS were searched as described above against transcriptome data available for mantle tissue of *M. edulis* 63 and *M*. *galloprovincialis* 57. Raw files were downloaded from the European Nucleotide Archive available on http://www.ebi.ac.uk/ena. Standard flowgram format (SFF) files of pyrosequencing from the 454 Life Sciences platform 63 were *de novo*‐assembled with the program gs de novo assembler (version 2.9) (Roche, Basel, Switzerland) according to the software manual instructions and under default parameters. Within the output files, the file 454Isotigs.faa (FASTA file of all ORFs of 100 bp or more found in each isotig nucleotide sequence) was used for MS/MS searches. A list of transcripts from *M*. *galloprovincialis* 57 was directly used for searches, or indirectly after being translated into six frames. Identified sequences were sent to BLAST 64. Only results with an *E* value < 1e–10 were reported. Open reading frames searches were carried out with the web tool from the NCBI (available on https://www.ncbi.nlm.nih.gov/orffinder/). Alignment of sequences was achieved with the Clustal Omega program available on the Uniprot website (http://www.uniprot.org/align/).

The mass spectrometry proteomics data have been deposited with the ProteomeXchange Consortium via the PRIDE 65 partner repository with the dataset identifier PXD004706.

## Data Accessibility

Data are available via ProteomeXchange [https://www.ebi.ac.uk/pride/archive/] with identifier PXD004706.

## Author contributions

JCI, AGF, and DPE conceived and designed the experiments and wrote the manuscript. JCI and DPE performed the experiments and contributed reagents/materials/analysis tools.
